# Circulating tumor cells as a prognostic biomarker in patients with hepatocellular carcinoma

**DOI:** 10.1038/s41598-022-21888-9

**Published:** 2022-11-04

**Authors:** Thaninee Prasoppokakorn, Areeya Buntho, Praewphan Ingrungruanglert, Thodsawit Tiyarattanachai, Tassanan Jaihan, Kittipat Kulkraisri, Darlene Ariyaskul, Chonlada Phathong, Nipan Israsena, Rungsun Rerknimitr, Sombat Treeprasertsuk, Roongruedee Chaiteerakij

**Affiliations:** 1 Division of Gastroenterology, Department of Medicine, Faculty of Medicine, Chulalongkorn University and King Chulalongkorn Memorial Hospital, Thai Red Cross Society, Bangkok, Thailand; 2 Center of Excellence in Stem Cell and Cell Therapy, Faculty of Medicine, Chulalongkorn University, Bangkok, Thailand; 3 Excellence Center for Stem Cell and Cell Therapy, King Chulalongkorn Memorial Hospital, Thai Red Cross Society, Bangkok, Thailand; 4 Faculty of Medicine, Chulalongkorn University, Bangkok, Thailand; 5 Center of Excellence for Innovation and Endoscopy in Gastrointestinal Oncology, Faculty of Medicine, Chulalongkorn University, 1873 Rama IV Road, Pathumwan, Bangkok, 10330 Thailand

**Keywords:** Liver cancer, Prognostic markers

## Abstract

Circulating tumor cells (CTCs) have been shown as a surrogate for cancer progression and prognostication. We aimed to determine an association between CTCs and survival of hepatocellular carcinoma (HCC) patients. Peripheral blood was obtained from 73 HCC patients to enumerate for epithelial CTCs/8 mL blood. CTCs were detected by immunoaffinity-based method using epithelial cell adhesion molecule (EpCAM) and mucin1 (MUC1). The CTCs detection rates of BCLC stages A, B, and C patients were 65.4% (17/26), 77.3% (17/22), and 96% (24/25), respectively, *p* = 0.018. Patients with CTCs < 5 cells/8 mL had significantly longer survival than those with CTCs ≥ 5 cells/8 mL (>36 vs. 4.6 months, *p* < 0.001). In multivariate analysis, CTP B, BCLC B, BCLC C, AFP ≥ 400 ng/mL, and CTC ≥ 5 cells/8 mL were independently associated with survival, with adjusted HRs (95%CI) of 4.1 (2.0–8.4), 3.5 (1.1–11.4), 4.7 (1.4–15.4), 2.4 (1.1–5.0), and 2.6 (1.2–8.4); *p* < 0.001, 0.036, 0.011, 0.025 and 0.012, respectively. The combination of CTCs ≥ 5 cells/8 mL and AFP ≥ 400 ng/mL provided additively increased HR to 5.3 (2.5–11.1), compared to HRs of 4.0 (2.0–8.0) and 3.5 (1.8–6.7) for CTCs ≥ 5 cells/8 mL and AFP ≥ 400 ng/mL, *p* < 0.001, respectively. The larger number of peripheral CTCs is correlated with higher tumor aggressive features and poorer survival of HCC patients. CTCs can potentially become novel prognostic biomarker in HCC.

## Introduction

Hepatocellular carcinoma (HCC) is one of the leading causes of cancer-related morbidity and mortality worldwide^1^. Currently, HCC can be treated with various treatment modalities at every stage of the disease^2,3^. However, the prognosis of HCC patients remains generally poor despite many treatment options available. One of the possible explanations for the poor prognosis is the increased resistance to treatments in advanced stage of HCC^4^. Additionally, the effectiveness of alpha-fetoprotein (AFP) which is the main biomarker for HCC used in clinical practice was limited due to the high heterogeneity of HCC^5^. This highlights the importance of HCC early detection in the hope to increase overall survival rate of HCC patients^6^. Therefore, novel effective biomarkers are urgently needed.

One of the advanced technologies increasingly applied for identifying novel cancer biomarkers is a liquid biopsy, which provides a wide range of molecular information in the blood circulation. Enumeration of circulating tumor cells (CTCs) is one of the information obtained from liquid biopsies. CTCs are neoplastic cells released from the primary tumor into the blood circulation and the presence of CTCs has shown to be fundamental to the cancer metastasis process^7,8^. Recently, enumeration of CTCs has been increasingly investigated for its potential in clinical practice such as being a criterion for selection of the first-line treatment in metastatic breast cancer^9^ or biomarker for monitoring treatment in patients with metastasis colorectal cancer^10^. Additionally, the detection of CTCs in peripheral blood may influence the treatment decision at an early stage of different types of cancer, particularly in prostate cancer^11^.

Regarding HCC, CTCs showed a potential for clinical applicability including disease staging^12^ and prognostication^13^. Most previous CTCs studies were conducted in cohorts of HCC patients treated with surgical resection. The ability of CTCs to prognosticate HCC recurrence after resection was consistently reported^14,15^. Few studies investigated an association between CTCs and survival of HCC patients. For example, Ogle et al. enrolled 69 HCC patients of whom 72% had Barcelona-Clinic Liver Cancer (BCLC) stage C HCC. The investigator found that patients with CTCs > 1 cell/4 mL blood had significant poor survival^16^.

Due to the limited data on performance of CTCs in predicting survival of HCC patients, we therefore researched to expand the knowledge with the primary aim to determine an association between baseline peripheral CTCs and patient survival. Our secondary aims were to: (1) determine an association between CTCs and HCC stages and aggressive features of HCC; and (2) determine the association between CTCs in combination with AFP as a predictor for survival of HCC patients.

## Materials and methods

### Study design and participants

The study protocol was approved by the Institutional Review Board of the Faculty of Medicine, Chulalongkorn University (IRB No.333/62), and was in accordance with the Helsinki Declaration of 1983. The study was registered in the Thai Clinical Trials Registry (TCTR20211226001) on 26th December 2021. All participants provided written informed consent prior to enrollment.

A single-center, prospective cohort study was conducted at the King Chulalongkorn Memorial Hospital, Bangkok, Thailand. Patients were enrolled between December 2018 and July 2020 and were followed up until December 2021. The inclusion criteria were newly diagnosed HCC patients aged ≥ 18 years. The exclusion criteria were patients with previous or concurrent malignancies and patients with mixed hepatocholangiocarcinoma. The diagnosis of HCC was made by typical radiologic findings in dynamic computed tomography or magnetic resonance imaging, or histology. HCC stages and severity of cirrhosis were determined by the Barcelona-Clinic Liver Cancer (BCLC) staging system; and Model for End-stage Liver Disease (MELD) score and Child-Turcotte-Pugh (CTP) class, respectively^2,3^. Baseline patient characteristics, HCC stage, underlying chronic liver diseases, presence of cirrhosis, severity of cirrhosis, and laboratory tests were abstracted from the electronic medical records. Peripheral blood of 8 mL was collected and measured for the number of CTCs at the outpatient unit during the initial visit or before any oncologic treatment. Subsequently, all patients received optimal treatment decided by a multidisciplinary team including hepatologists, surgeons, interventionists, and oncologists. All patients were followed for disease progression and vital status.

**Figure 1:**
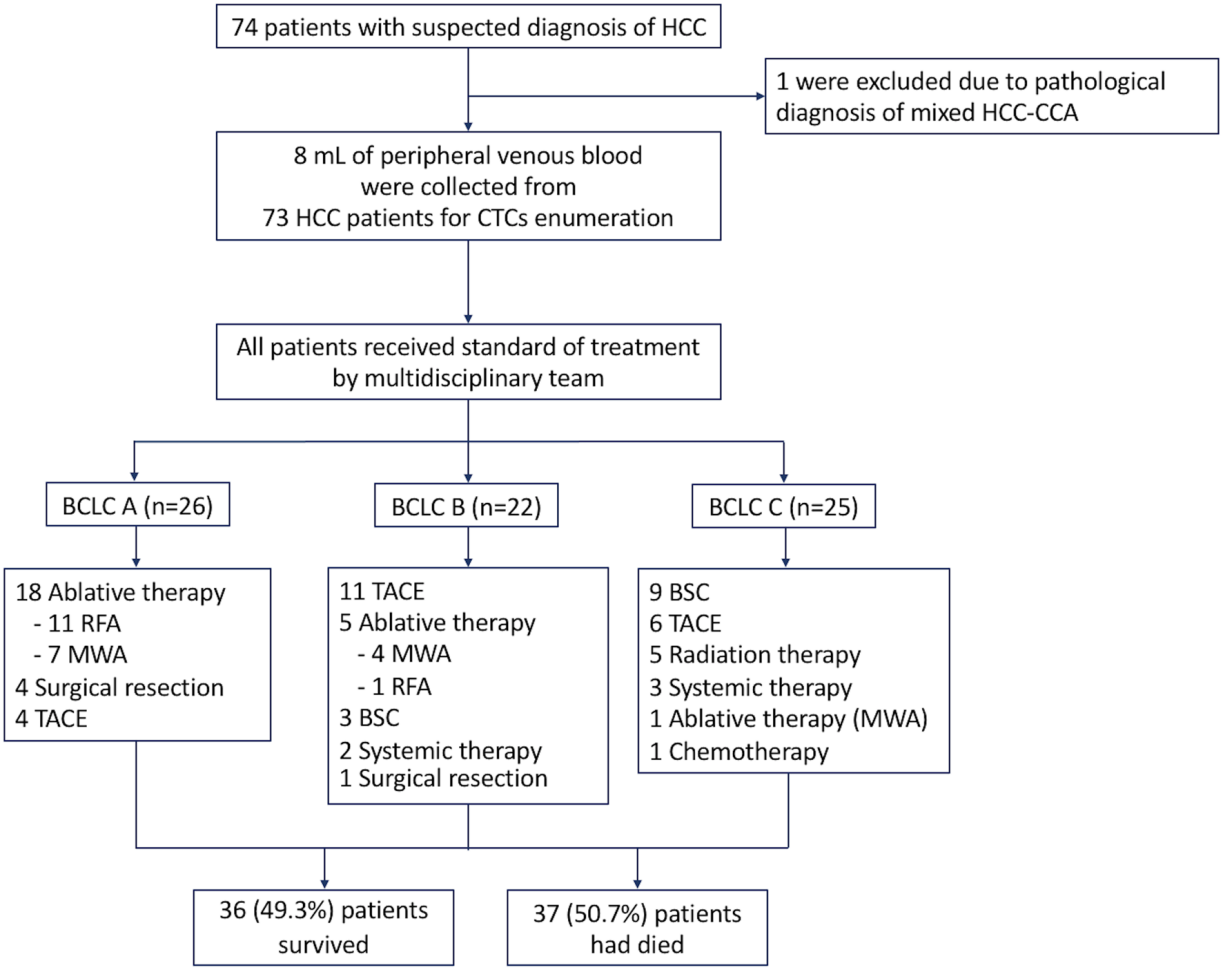
Flow diagram of patient enrollment. Abbreviations: BCLC; Barcelona clinic liver cancer. BSC; best supportive care. CTCs; circulating tumor cells. HCC-CCA; hepato-cholangiocarcinoma. HCC; hepatocellular carcinoma. MWA; microwave ablation. RFA; radiofrequency ablation. TACE; transarterial chemoembolization.

In this study, tumor aggressive features were defined as a tumor with the size of ≥ 5 cm, ≥ 3 nodules, vascular invasion, lymph node involvement, and/or distant metastasis. In our institution, HCC patients with BCLC stage A, B, and C received surgical resection, transarterial chemoembolization (TACE), and systemic therapy as an initial treatment, respectively. However, if the initial treatment modality was not feasible, other available treatments were selected as appropriate for each patient (Fig. 1).

### CTCs enumeration

Eight mL of whole blood was drawn from the peripheral vein of patients and collected in preservative tubes containing heparin. The CTC enumeration was performed within 4 h after blood sampling. Immunoaffinity-based method for CTC detection relies on the epithelial-specific antibodies against 2 cell surface markers: epithelial cell adhesion molecule (EpCAM, CD326) and mucin 1 (MUC1). CTC enumeration was performed using magnetic separation, fluorescence antibody staining, and PerkinElmer Opera Phenix™ | High Content Screening System^17,18^. The details of CTC enumeration process were provided in the Supplemental method^19^. CTCs were counted if the cells had positive staining for EpCAM and MUC1 and reported as number of cells per 8 mL of venous blood (Supplemental Figure).

### Statistical analysis

Continuous variables were presented as mean ± standard deviation (SD) or median (interquartile range, IQR) and compared using unpaired Student’s t-tests, the Mann–Whitney U test, the Mood’s median test, or one-way ANOVA test as appropriate. Categorical variables were presented as number (percent) and compared using Fisher’s exact test or Chi-square test as appropriate. The optimal cutoff of CTCs was the value which provided the best sensitivity and specificity determined using the area under the receiver operating characteristics (AUROCs). Subgroup analysis by treatment modality was analyzed by using the optimal cutoff of CTCs. The prognostic cutoff of AFP at ≥ 400 ng/mL was determined as an effective cut point for HCC based on the findings from previous literatures^20,21^. In addition, the cutoff of Model for End-stage Liver Disease (MELD) at ≥ 15 was also determined for prognostication.

Patient survival was calculated from enrollment date to death date or last follow up date. The correlation between number of CTCs and patient survival was analyzed by using coefficient of correlation (r). Overall median survival was estimated using the Kaplan–Meier method. The survivals of patients were classified as BCLC stages and CTP class and compared using the Log Rank test. An association between CTCs and patient survival was determined using Cox proportional hazards analysis. Other factors associated with patient survival were also determined using the univariate Cox proportional hazards model. Age, sex, and other factors with *p* value of < 0.05 in the univariate model were included in the multivariate model. Statistical analyses were performed using the SPSS package version 22.0.0 (SPSS Inc., Chicago, Illinois, USA). A *p* value of < 0.05 was considered statistically significant.

## Results

### Baseline patient characteristics

Of the total of 73 HCC patients, 56 (76.7%) were males with a mean ± SD age of 60.2 ± 12.0 years. Most patients (n = 50, 68.5%) had CTP class A cirrhosis. The mean ± SD of the tumor size was 6.0 ± 4.7 cm and 33 (45.2%) patients had tumor ≥ 3 nodules. Baseline characteristics of the entire cohort are displayed in Table 1.

**Table 1:** Baseline characteristics of HCC patients in the study cohort.

Variables	Total	BCLC A	BCLC B	BCLC C	*p*
	(n = 73)	(n = 26)	(n = 22)	(n = 25)	
Age (y)*	60.2 ± 12.0	61.0 ± 11.2	60.7 ± 10.1	58.8 ± 14.4	0.759
Male, n(%)	56 (76.7%)	20 (76.9%)	19 (86.4%)	17 (68.0%)	0.331
**Cirrhosis class, n(%)**					0.64
CTP A	50 (68.5%)	22 (84.6%)	14 (63.6%)	14 (56.0%	
CTP B	23 (31.5%)	4 (15.4%)	8 (36.4%)	11 (44.0%)	
**Etiology of cirrhosis, n(%)**					0.127
HBV	27 (37.0%)	6 (23.1%)	8 (36.4%)	13 (52.0%)	
HCV	22 (30.0%)	10 (38.5%)	5 (22.7%)	7 (28.0%)	
Alcohol	8 (11.0%)	4 (15.4%)	3 (13.7%)	1 (4.0%)	
NASH	8 (11.0%)	3 (11.5%)	5 (22.7%)	0 (0.0%)	
Cryptogenic	8 (11.0%)	3 (11.5%)	1 (4.5%)	4 (16.0%)	
**Tumor number, n(%)**					0.012
1	26 (35.6%)	14 (53.9%)	4 (18.2%)	8 (32.0%)	
2	14 (19.2%)	7 (26.9%)	3 (13.6%)	4 (16.0%)	
≥ 3	33 (45.2%)	5 (19.2%)	15 (68.2%)	13 (52.0%)	
Tumor size (cm)*	6.0 ± 4.7	2.6 ± 1.4	6.0 ± 4.3	9.3 ± 4.8	< 0.001
Globulin (g/dL)*	4.3 ± 1.0	4.0 ± 0.8	4.4 ± 0.9	4.6 ± 1.3	0.029
Albumin (g/dL)*	3.7 ± 0.7	4.0 ± 0.6	3.5 ± 0.7	3.4 ± 0.5	0.001
TB (mg/dL)*	1.5 ± 1.4	0.9 ± 0.4	1.3 ± 0.8	2.2 ± 2.1	0.004
DB (mg/dL)*	0.8 ± 1.1	0.4 ± 0.3	0.7 ± 0.5	1.3 ± 1.6	0.003
AST (U/L)*	91 ± 123	40 ± 36	94 ± 143	140 ± 145	< 0.001
ALT (U/L)*	50 ± 60	37 ± 35	64 ± 94	52 ± 36	0.003
ALP (U/L)*	138 ± 80	95 ± 36	123 ± 43	196 ± 103	< 0.001
AFP (ng/mL) median(IQR)	36 (5404)	7 (3142)	22 (5401)	340 (340,22,291)	0.003
AFP ≥ 400 ng/mL, n(%)	21 (28.8%)	2 (7.7%)	6 (27.3%)	13 (52.0%)	0.001
MELD score*	10.9 ± 4.1	9.9 ± 4.5	10.4 ± 2.3	12.3 ± 4.5	0.062
CTCs detection, n(%)	58 (79.5%)	17 (65.4%)	17 (77.3%)	24 (96.0%)	0.018
CTCs (cells/8 mL) median (IQR)	4.0 (1.0,11.0)	1.5 (0.0, 4.0)	3.5 (0.8, 13.3)	6.0 (3.5, 37.5)	0.002
Deceased, n(%)	37 (50.7%)	4 (15.4%)	12 (54.5%)	21 (84.0%)	< 0.001
Median survival (months)	20.1	22.5	15.6	5.6	< 0.001

Data with * are presented as mean ± standard deviation.

*AFP* Alpha fetoprotein, *ALBI* Albumin-bilirubin score, *ALP* Alkaline phosphatase, *ALT* Alanine aminotransferase, *AST* Aspartate transaminase, *BCLC* Barcelona clinic liver cancer, cm Centimeter, *CTCs* Circulating tumor cells, *CTP* Child-Turcotte-Pugh score, *DB* Direct bilirubin, *g/dL* Gram per deciliter, *HBV* Hepatitis B virus, *HCV* Hepatitis C virus, *IQR* Interquartile range, *MELD* Model for end-stage liver disease. *NA* Not applicable, *ng/mL* Nanogram per milliliter, *NASH* Nonalcoholic steatohepatitis, *SD* Standard deviation, *TB* Total bilirubin, *U/L* Units per liter.

There were 26 (35.6%), 22 (30.1%), and 25 (34.3%) patients in the BCLC stages A (early), B (intermediate), and C (advanced), respectively (Table 1). The tumor size was significantly different among the 3 stages, with the sizes of 2.6 ± 1.4, 6.0 ± 4.3, and 9.3 ± 4.8, for BCLC stages A, B, and C, respectively, *p* < 0.001. The degree of liver impairment was significantly different among the 3 groups, with the most impairment in the BCLC C group, followed by BCLC B and A groups, respectively. The severity of liver impairment was characterized by a decrease in serum albumin level (4.0 ± 0.6 vs. 3.5 ± 0.7 vs. 3.4 ± 0.5 g/dL, p = 0.001) and increase in total bilirubin level (0.9 ± 0.4 vs. 1.3 ± 0.8 vs. 2.2 ± 2.1 mg/dL, *p* = 0.004) across BCLC A, B, C group, respectively. The median AFP levels were highest in the BCLC stage C patients, followed by BCLC stage B, and A patients, respectively (340 vs. 22 vs. 2 ng/mL, *p* = 0.003).

Of the 26 patients with BCLC stage A, 18 (69.2%) patients received ablative therapy (11 radiofrequency ablation and 7 microwave ablation), while 4 (15.4%) patients received surgical resection and 4 (15.4%) received TACE. In BCLC stage B group, half received TACE (n = 11, 50.0%), followed by ablative therapy (n = 5, 22.7%) and best supportive care (n = 3, 13.6%). Patients with BCLC stage C received best support care (n = 9, 36.0%), TACE (n = 6, 24.0%), and radiation therapy (n = 5, 20.0%). Details of treatment options for patients with each BCLC stage are provided in Fig. 1.

### Enumeration of CTCs and HCC aggressive features

The CTCs were detected in the peripheral blood of 58 (79.5%) patients. The detection rate was significantly different among HCC stages, with 65.4% (17/26), 77.3% (17/22), and 96% (24/25) for BCLC stages A, B, and C, respectively, *p* = 0.018. The mean number of CTCs of the entire cohort was 21.3 ± 84.3 cells/8 mL. Likewise, the number of detected CTCs were significantly different among the 3 stages, with increasing CTCs numbers in the more advanced stages with medians of 1.5, 3.5, and 6.0 cells for stages A, B, and C, *p* = 0.002, respectively.

Patients with BCLC stage C had significantly higher median CTCs than those with stages A/B (6.0 vs. 2.0 cells, *p* = 0.006). Likewise, patients with vascular invasion had significantly greater CTCs than those without vascular invasion (6.0 vs. 2.0 cells, *p* = 0.024). Patients with other aggressive HCC features, including larger tumor size, tumor number ≥ 3, lymph node involvement, and distant metastasis also had more CTCs than those without these features (Table 2).

**Table 2:** Comparison of CTCs number between HCC patients with and without aggressive features.

Variables	Number of CTCs/8 mL of blood (median, interquartile range)*		*p*
Tumor size	Size < 5 cm (n = 40)	Size ≥ 5 cm (n = 33)	0.017
	2.0 (0.0, 5.0)	6.0 (2.0, 16.0)	
Tumor number	Number < 3 (n = 40)	Number ≥ 3 (n = 33)	0.094
	2.0 (1.0, 5.0)	6.0 (1.5, 19.0)	
Vascular invasion	No (n = 48)	Yes (n = 25)	0.024
	2.0 (0.0, 5.8)	6.0 (2.5, 25.0)	
Lymph node metastasis	No (n = 62)	Yes (n = 11)	0.269
	2.5 (1.0, 9.3)	6.0 (3.0, 40.0)	
Distant metastasis	No (n = 69)	Yes (n = 4)	0.439
	3.0 (1.0, 10.5)	80.5 (4.5, 557.8)	
BCLC stage of HCC	A and B (n = 48)	C (n = 25)	0.006
	2.0 (0.0, 5.0)	6.0 (3.5, 37.5)	

*CTCs numbers were not normally distributed, we used the Mann–Whitney U test to compare the differences in number of CTCs between patients with and without HCC aggressive features.

### Performance of CTCs for predicting HCC aggressive features

**Figure 2:**
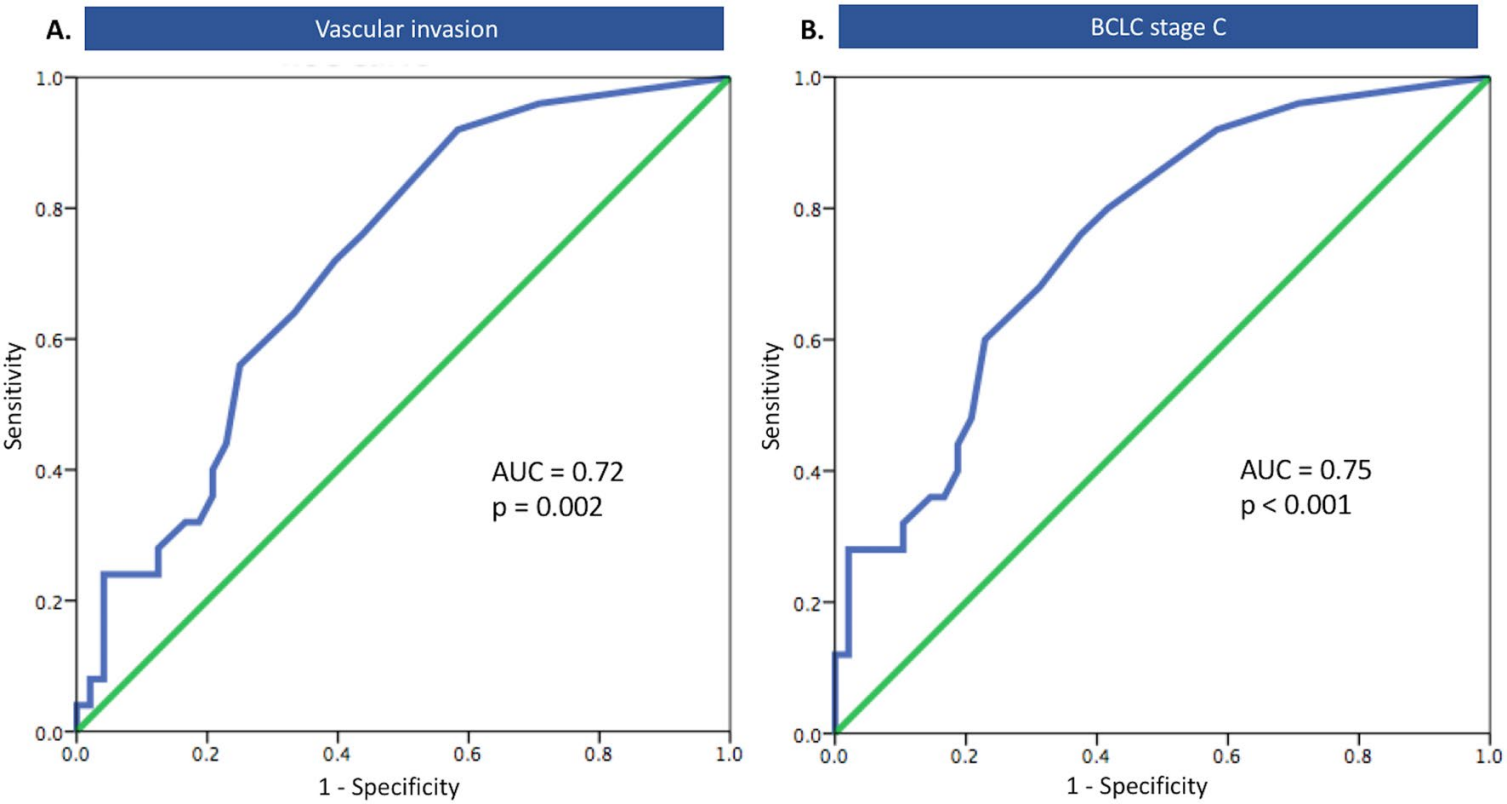
The AUROC curve of peripheral CTCs for prediction of vascular invasion (**A**), and BCLC stage C (**B**).

The number of CTCs predicted the aggressive features of HCC, with AUROCs of 0.72 (95%CI: 0.60–0.84, *p* = 0.002) for vascular invasion, and 0.75 (95%CI: 0.64–0.87, *p* < 0.001) for BCLC stage C, respectively (Fig. 2A–B). Using a cutoff of ≥ 5 cells/8 mL blood, CTCs differentiated HCC patients with vascular invasion from patients who did not have vascular invasion with a sensitivity and specificity of 64.0 and 66.7%, respectively. The sensitivity and specificity of CTCs of ≥ 5 cells/8 mL for predicting BCLC stage C were 68.0 and 68.7%, respectively.

### Association between CTCs number and survival of HCC patients

**Figure 3:**
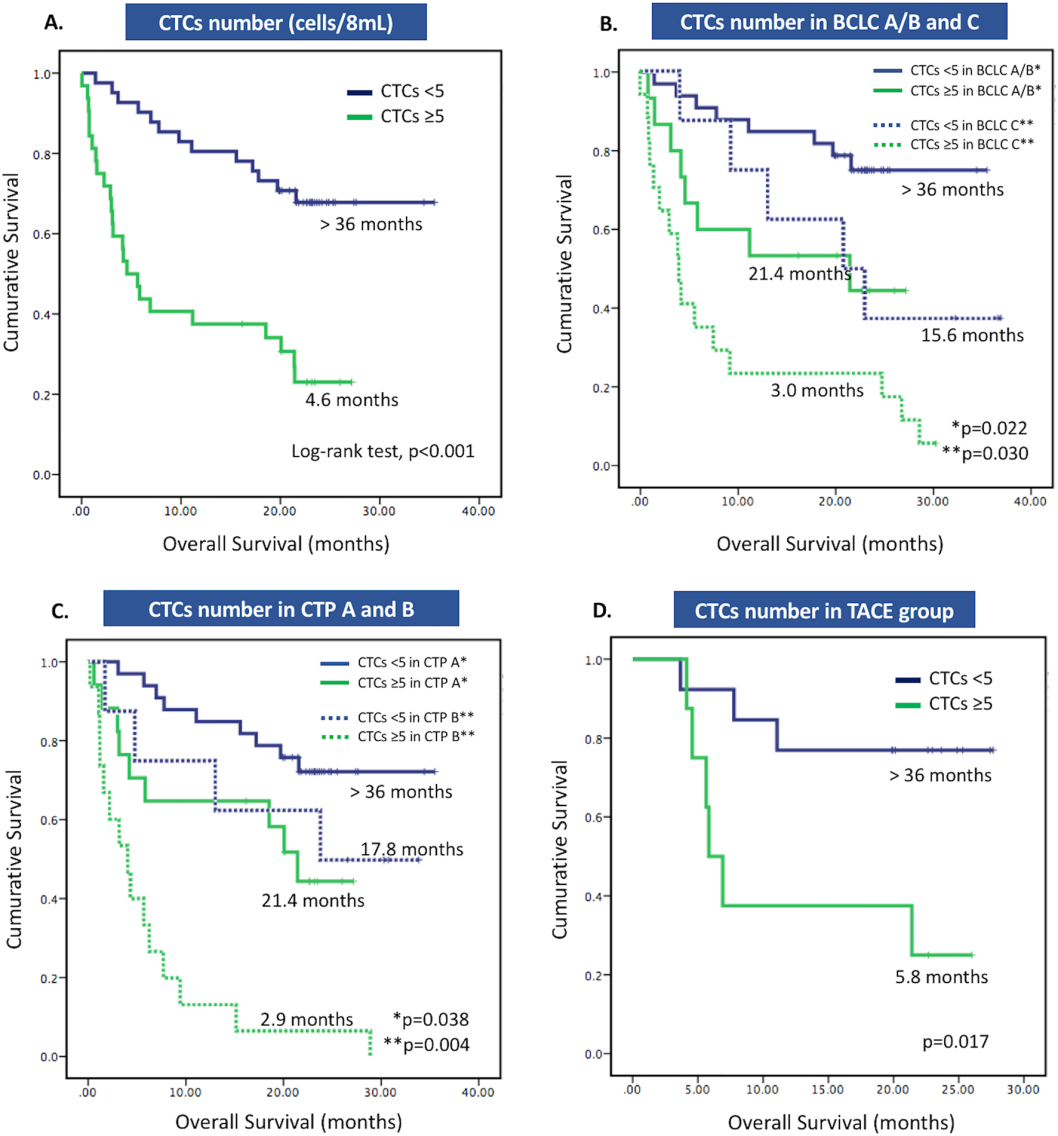
Survival of HCC patients classified by the number of CTCs (**A**), BCLC stages (**B**), and Child-Turcotte-Pugh (CTP) classes (**C**), and survival of HCC patients treated with TACE (**D**).

During the follow-up period of 36.0 months, 37 (50.7%) patients had died. The median survival of the entire cohort was 21.1 months. The number of CTCs had a significant inverse correlation with patient survival (r = − 0.288, *p* = 0.014). The deceased patients had a significantly higher number of CTCs than those who survived, with median CTCs of 6.0 versus 2.0 cells, *p* = 0.001. Patients with CTCs < 5 cells/ 8 mL had significantly longer median survival than those with CTCs ≥ 5 cells/8 mL (> 36.0 vs. 4.6 months, *p* < 0.001) (Fig. 3A). When classified patients by BCLC stages, the finding remained consistent. The median survival for patients with CTCs < 5 cells/8 mL remained significantly longer for both combined BCLC A/B subgroup (> 36 vs. 21.4 months, *p* = 0.022) and BCLC C subgroup (15.6 vs. 3.0 months, *p* = 0.030) (Fig. 3B), respectively. Likewise, when categorized by the degree of liver impairment, patients with CTCs < 5 cells/8 mL also had significantly longer survival than those with CTCs ≥ 5 cells/8 mL.

The similar findings were found when classified patients by CTP system. In the CTP B (decompensated) cirrhosis subgroup, the survivals of patients with CTCs < 5/8 mL and CTCs ≥ 5 cells/8 mL were 17.8 versus 2.9 months, respectively, *p* = 0.004. However, in CTP A (compensated) cirrhosis subgroup, the estimated median survival of patients with CTCs < 5 cells/8 mL was not reached at a median follow up of 22.7 months, while those with CTCs ≥ 5 cells/8 mL had a median survival of 21.4 months, *p* = 0.038 (Fig. 3C).

Regarding treatment modality, TACE was the most frequently used in this cohort (n = 21), hence we opted to perform the subgroup analysis of patients treated with TACE. In the TACE subgroup, patients with CTCs < 5 cells/8 mL had significantly longer survival than those with CTCs ≥ 5 cells/8 mL (> 36.0 vs. 5.8 months, *p* = 0.017) (Fig. 3D).

In the univariate analysis, CTCs ≥ 5 cells/8 mL were significantly associated with decreased survival with hazard ratio (HR) (95%CI) of 4.04 (2.04–7.99, *p* < 0.001). Other factors significantly associated with survival included CTP B, BCLC stages B and C, and AFP ≥ 400 ng/mL, (Table 3). After adjusted for age and sex in multivariate analysis, The CTP B, BCLC B, and C stages were also independently associated with worse survival with adjusted HR of 4.11 (95%CI: 2.02–8.39, *p* < 0.001), 3.52 (95%CI: 1.09–11.36, *p* = 0.036), and 4.67 (95%CI 1.41–15.43, *p* = 0.011), respectively. CTCs ≥ 5 cells/8 mL, and AFP ≥ 400 ng/mL remained significantly associated with survival with an adjusted HR 2.61 (95%CI: 1.23–8.39, *p* =  0.012), 2.36 (95%CI: 1.12–5.00, *p* = 0.025), respectively. The combination biomarkers of CTCs ≥ 5 and AFP ≥ 400 ng/mL provided adjusted HR 2.58 (95%CI: 1.15–5.80, *p* = 0.022) (Table 3).

**Table 3:** Univariate and multivariate Cox regression analysis of factors associated with overall survival of HCC patients.

Variables	Univariate analysis		Multivariate analysis	
	HR	*p*	Adjusted HR	*p*
	(95%CI)			
Age (years)	1.00 (0.97–1.02)	0.69	(95%CI)*	
**Sex**				
Male	1 (reference)			
Female	1.11 (0.77–1.59)	0.58		
CTP A	1 (reference)		1 (reference)	
CTP B	4.45 (2.30–8.59)	< 0.001	4.11 (2.02–8.39)	< 0.001
BCLC A	1(reference)			
BCLC B	5.06 (1.63–15.73)	0.005	3.52 (1.09–11.36)	0.036
BCLC C	11.00 (3.75–32.32)	< 0.001	4.67 (1.41–15.43)	0.011
AFP ≥ 400 ng/mL	3.45 (1.78–6.69)	< 0.001	2.36 (1.12–5.00)	0.025
CTCs ≥ 5 cells/8 mL	4.04 (2.04–7.99)	< 0.001	2.61 (1.23–8.39)	0.012
CTCs ≥ 5 cells/8mL and AFP ≥ 400 ng/mL	5.29 (2.52–11.09)	< 0.001	2.58 (1.15–5.80)	0.022

*Adjusted by age and sex.

### Association between combined CTCs and AFP level and survival of HCC patients

**Figure 4:**
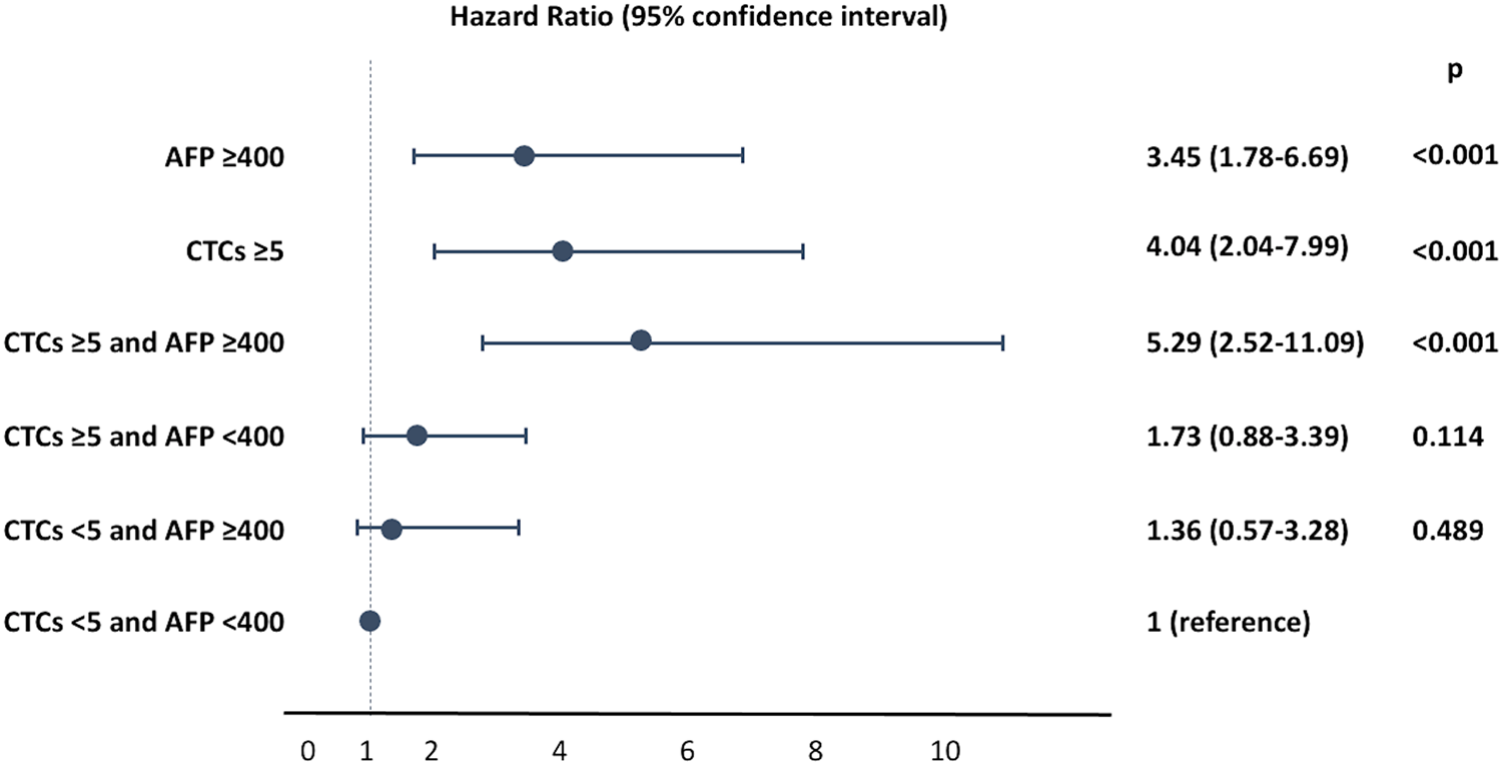
Association between combinations of CTCs and AFP and survival of HCC patients.

Next, we determined the effectiveness of combined CTCs and AFP as a predictor for survival of HCC patients. We found that CTCs ≥ 5 cells/8 mL and AFP ≥ 400 ng/mL were significantly associated with patient survival, with HRs of 4.04 (95%CI 2.04–7.99, *p* < 0.001) and 3.45 (95%CI 1.78–6.69, *p* < 0.001), respectively. When CTCs and AFP were combined, the HR additively increased to 5.29 (95%CI 2.52–11.09, *p* < 0.001), supporting that the combination of biomarkers was more useful in predicting outcomes of patients than a single biomarker (Fig. 4).

## Discussion

We demonstrated the potential of CTCs as a tumor biomarker for predicting HCC aggressive features and survival of HCC patients. More CTCs were detected in the peripheral blood of patients with more advanced HCC stages. The number of CTCs was also independently associated with patient survival. Accordingly, peripheral CTCs measurement may offer more prognostic information for HCC patients.

In this study, the number of CTCs was significantly greater in HCC patients with more aggressive features. This positive correlation between CTCs and HCC aggressive phenotypes were consistent with previous study reporting that the CTCs number significantly correlated with the tumor size and the presence of portal vein thrombosis^16^. In another group of 73 HCC patients treated with surgical resection, the number of CTCs ≥ 2 cells in peripheral venous blood was reported to be associated with microvascular invasion and intrahepatic recurrence after tumor resection^22^. Our findings and previous studies consistently indicated that CTCs may be a surrogate marker for HCC aggressive features.

Of note, the CTCs were detectable since the early stage of HCC when the metastasis in distant organs cannot be visualized by radiologic imaging^7,13^. Therefore, peripheral CTCs could have the potential to serve as biomarkers for early detection of metastasis. Clinically, this could have implications in monitoring the disease after therapy and tailoring treatment strategies for patients with a high number of CTCs. These patients may require more frequent and intensive follow-up imaging after treatment to promptly detect metastasis to prevent disease recurrence.

Our results suggested that there was an inverse correlation between number of CTCs and survival of HCC patients across all BCLC stages of the disease. These findings were consistent with previous reports^12,23^. We also found that the CTCs load was useful in predicting survival of patients with both compensated (CTP A) and decompensated (CTP B) cirrhosis. Patients with CTP A cirrhosis with low CTCs load had the best survival, while patients with CTP B cirrhosis with high CTCs load had the worst survival. Because our study included HCC patients from different disease stages and cirrhosis severity, it was possible for us to establish the association between number of CTCs and patient survival from multiple HCC conditions. Our results showed a consistent pattern, supporting that higher number of CTCs were correlated with worse patient survival in every HCC condition. These findings suggested that peripheral CTCs level can be a strong predictor of survival in HCC patients regardless of their disease stages and cirrhosis severity.

Although multiple prognostic scoring systems or prediction models have been proposed for determining HCC prognosis^24^, AFP remains one of the biomarkers most commonly used for HCC prognosis albeit its many limitations^25^. Interestingly, previous studies found that increased level of AFP in HCC patients were correlated with higher number of CTCs^26,27^. In our study, we proposed a novel prognostic tool by using the combination of CTCs and AFP as survival predictors for HCC patients. The HR of combined CTCs and AFP was higher than HR of CTCs or AFP alone. This suggested that the combination of biomarkers was an effective HCC prognostic tool and worth further investigation.

This study had several strengths. We found that AFP and CTCs together were a more effective predictor of patient survival than individual measurement, suggesting a novel prognostic tool for HCC patients. Furthermore, this study included patients with multiple stages of HCC, which provided samples that can represent HCC patients at different courses of disease development. This indicated that our results of CTCs as prognostic markers may be generalizable to most HCC patients and allowed them to receive early detection of metastasis and preemptive treatment. This study had some limitations. Firstly, we counted only the number of epithelial CTCs and did not isolate CTCs with other phenotypes, such as mesenchymal CTCs and mixed epithelial-mesenchymal CTCs. Second, we measured the number of CTCs at a single time point. Serial measurement would provide more information to better prognosticate patient outcomes. Thirdly, after performing liquid biopsies, patients had received different treatment modalities, which could possibly impact on the patients’ health outcomes. To minimize the impact of different treatment modalities on the prognostic ability of CTC on patient survival, we performed a subgroup analysis of patients who received TACE, the finding remained consistent with the main analysis. Thus, for our next prospective study, we planned to recruit more homogeneous HCC cohorts (i.e. focusing on each treatment modality) in order to demonstrate the add-on benefit of CTCs in the management of HCC patients.

## Conclusion

The increment of peripheral CTCs is associated with more HCC aggressive features and poor survival of HCC patients. CTCs enumeration has the potential to be used as an additional prognostic biomarker in HCC.

## Supplementary Information

Supplementary Information.

## Data Availability

All data generated or analyzed during this study are available upon request.

## References

[1] Sayiner M, Golabi P, Younossi ZM (2019). Disease burden of hepatocellular carcinoma: A global perspective. Dig. Dis. Sci..

[2] European Association for the Study of the Liver (2018). EASL clinical practice guidelines: Management of hepatocellular carcinoma. J. Hepatol..

[3] Marrero JA, Kulik LM, Sirlin CB, Zhu AX, Finn RS, Abecassis MM (2018). Diagnosis, staging, and management of hepatocellular carcinoma: 2018 Practice guidance by the American association for the study of liver diseases. Hepatology.

[4] Guo J, Li L, Guo B, Liu D, Shi J, Wu C (2018). Mechanisms of resistance to chemotherapy and radiotherapy in hepatocellular carcinoma. Transl. Cancer Res..

[5] Chaiteerakij R, Addissie BD, Roberts LR (2015). Update on biomarkers of hepatocellular carcinoma. Clin. Gastroenterol Hepatol..

[6] Wang W, Wei C (2020). Advances in the early diagnosis of hepatocellular carcinoma. Genes Dis..

[7] Eslami SZ, Cortes-Hernandez LE, Alix-Panabieres C (2020). The metastatic cascade as the basis for liquid biopsy development. Front. Oncol..

[8] Pantel K, Speicher MR (2016). The biology of circulating tumor cells. Oncogene.

[9] Zhang H, Lin X, Huang Y, Wang M, Cen C, Tang S (2021). Detection methods and clinical applications of circulating tumor cells in breast cancer. Front. Oncol..

[10] Christou N, Meyer J, Popeskou S, David V, Toso C, Buchs N (2019). Circulating tumour cells, circulating tumour DNA and circulating tumour miRNA in blood assays in the different steps of colorectal cancer management, a review of the evidence in 2019. Biomed. Res. Int..

[11] Pantel K, Hille C, Scher HI (2019). Circulating tumor cells in prostate cancer: From discovery to clinical utility. Clin. Chem..

[12] Chen J, Cao SW, Cai Z, Zheng L, Wang Q (2017). Epithelial-mesenchymal transition phenotypes of circulating tumor cells correlate with the clinical stages and cancer metastasis in hepatocellular carcinoma patients. Cancer Biomark..

[13] Wang P-X, Cheng J-W, Yang X-R (2020). Detection of circulating tumor cells in hepatocellular carcinoma: Applications in diagnosis, prognosis prediction and personalized treatment. Hepatoma Res..

[14] Sun YF, Wang PX, Cheng JW, Gong ZJ, Huang A, Zhou KQ (2020). Postoperative circulating tumor cells: An early predictor of extrahepatic metastases in patients with hepatocellular carcinoma undergoing curative surgical resection. Cancer Cytopathol..

[15] Chen VL, Xu D, Wicha MS, Lok AS, Parikh ND (2020). Utility of liquid biopsy analysis in detection of hepatocellular carcinoma, determination of prognosis, and disease monitoring: A systematic review. Clin. Gastroenterol. Hepatol..

[16] Ogle LF, Orr JG, Willoughby CE, Hutton C, McPherson S, Plummer R (2016). Imagestream detection and characterisation of circulating tumour cells—A liquid biopsy for hepatocellular carcinoma?. J. Hepatol..

[17] Castro-Giner F, Aceto N (2020). Tracking cancer progression: from circulating tumor cells to metastasis. Genome Med..

[18] Habli Z, AlChamaa W, Saab R, Kadara H, Khraiche ML (2020). Circulating tumor cell detection technologies and clinical utility: Challenges and opportunities. Cancers (Basel).

[19] Takao M, Takeda K (2011). Enumeration, characterization, and collection of intact circulating tumor cells by cross contamination-free flow cytometry. Cytom. A.

[20] Kelley RK, Meyer T, Rimassa L, Merle P, Park JW, Yau T (2020). Serum alpha-fetoprotein levels and clinical outcomes in the phase III CELESTIAL study of cabozantinib versus placebo in patients with advanced hepatocellular carcinoma. Clin. Cancer Res..

[21] Tangkijvanich P, Anukulkarnkusol N, Suwangool P, Lertmaharit S, Hanvivatvong O, Kullavanijaya P, Poovorawan Y (2000). Clinical characteristics and prognosis of hepatocellular carcinoma analysis based on serum alpha-fetoprotein levels. J. Clin. Gastroenterol..

[22] Sun YF, Guo W, Xu Y, Shi YH, Gong ZJ, Ji Y (2018). Circulating tumor cells from different vascular sites exhibit spatial heterogeneity in epithelial and mesenchymal composition and distinct clinical significance in hepatocellular carcinoma. Clin. Cancer Res..

[23] Yu JJ, Shu C, Yang HY, Huang Z, Li YN, Tao R (2021). The presence of circulating tumor cell cluster characterizes an aggressive hepatocellular carcinoma subtype. Front. Oncol..

[24] Liu PH, Hsu CY, Hsia CY, Lee YH, Su CW, Huang YH (2016). Prognosis of hepatocellular carcinoma: Assessment of eleven staging systems. J. Hepatol..

[25] Hanif H, Ali MJ, Susheela AT, Khan IW, Luna-Cuadros MA, Khan MM (2022). Update on the applications and limitations of alpha-fetoprotein for hepatocellular carcinoma. World J. Gastroenterol..

[26] Ou H, Huang Y, Xiang L, Chen Z, Fang Y, Lin Y (2018). Circulating tumor cell phenotype indicates poor survival and recurrence after surgery for hepatocellular carcinoma. Dig. Dis. Sci..

[27] Yin LC, Luo ZC, Gao YX, Li Y, Peng Q, Gao Y (2018). Twist expression in circulating hepatocellular carcinoma cells predicts metastasis and prognoses. Biomed. Res. Int..

