# Supplementary material for: Circulating tumor cells as a prognostic biomarker in patients with hepatocellular carcinoma
Source: Sci Rep. 2022 Nov 4;12:18686. doi: 10.1038/s41598-022-21888-9 (PMC9636215; doi:10.1038/s41598-022-21888-9)
Supplement: Supplementary file 1 — Supplementary Information. [file 41598_2022_21888_MOESM1_ESM.docx]

**Supplemental method**

**Process of CTC enumeration**

The CTC enumeration was performed as follows: 1) whole blood samples were processed by diluted blood 1:1 with phosphate-buffered saline (PBS), 2) diluted blood was overlay on Ficoll-Paque^TM^ with a ratio of 4:3 and then centrifuged at 400 g 25°C for 35 minutes, 3) peripheral blood mononuclear cells (PBMCs) layer were carefully aspirated from the supernatant layer and centrifuged at 400g 20°C for 10 minutes, 4) the cell pellet was re-suspended with PBS and centrifuged 400 g at 20°C for 10 minutes; and the supernatant was discarded completely, 5) the cell pellet was re-suspended with staining buffer and centrifuged 300 g at 20°C for 10 minutes; and the supernatant was discarded completely. Subsequently, the process of magnetic labeling was executed as follows; 1) washed cells by adding staining buffer, 2) added FcR blocking reagent, mixed well and incubated for 10 minutes at room temperature, 3) added CD326 (epithelial cell adhesion molecule; EpCAM) Microbeads and incubated for 30 minutes at 4°C, 4) washed cells by adding staining buffer and centrifuged 300 g for 10 minutes, 5) resuspended cell pellet in buffer. Afterwards, magnetic separation was performed with MS columns as follows: 1) collected unlabeled cells which passed through and washed with buffer sequentially once the column reservoir was empty, 2) repeated the procedure for three times and collected total effluent as unlabeled cell fraction, 3) pipetted cool staining buffer onto the column, followed by immediately flush out fraction with magnetically labeled cells by firmly applying the plunger supplied with the column. Later, for immunofluorescence staining, CD326-positive cell filtrated were incubated with an anti-EpCAM antibody, anti- mucin 1 (MUC1), and anti-CD45 antibody in the presence of DNA staining dye Hoechst at 4°C in the dark for 30 minutes, followed by washing and centrifugation at 300 g for 5 minutes. Finally, CTCs detection base on intact nucleate epithelial cell, expression of EpCAM and MUC1, and negative for the leukocytes marker CD45 on Confocal microscope (Opera phenix) **(Supplemental Figure)**.

**
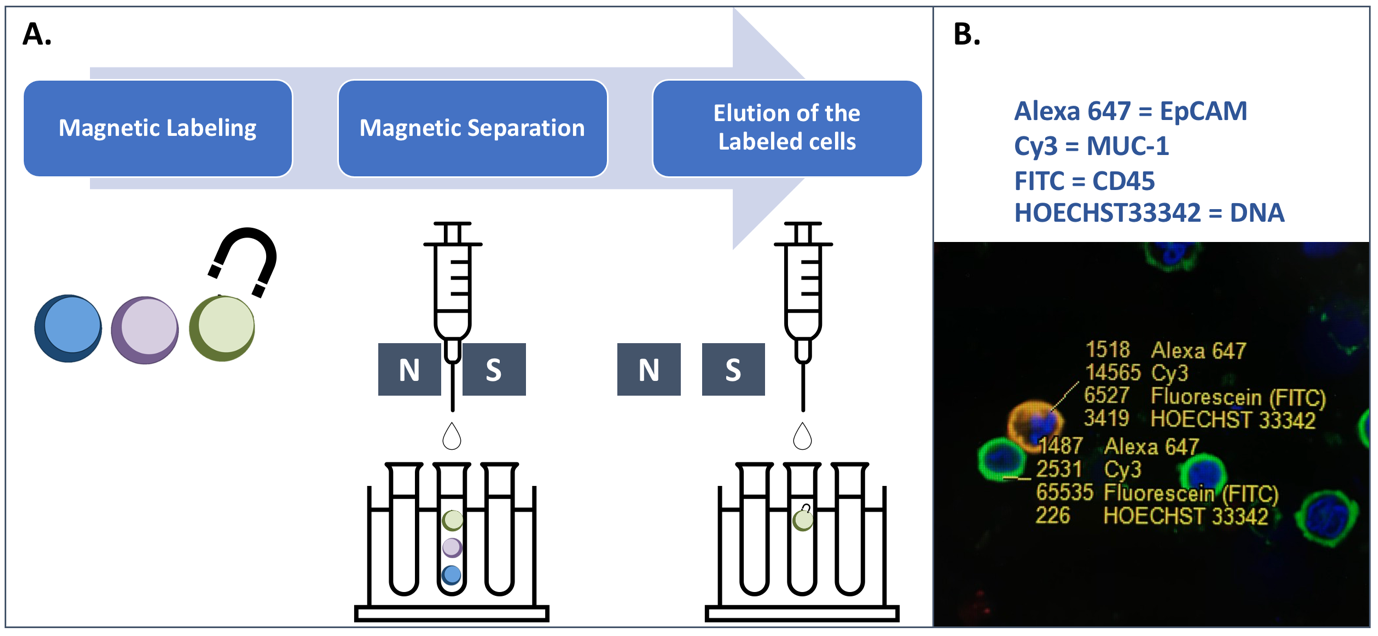
**

**Supplemental Figure.** CTCs enumeration by magnetic labeling and separation (A). Cells were counted as CTCs if positive for EpCAM and MUC1 and negative for CD45 (orange-colored cell) and white blood cells were cells positive for CD45 (green-color cell) on fluorescent microscopy (B).

CTC: circulating tumor cells, EpCAM: epithelial cell adhesion molecule, MUC1: mucin 1.
